# Advertisements

**Published:** 1884-03

**Authors:** 


### ADVERTISEMENTS.

We do not propose to admit to the advertising pages of the INDEPENDENT PRACTITIONER any announcement of an article that we have not either personally tried or examined, or that does not come to us with the endorsement of impartial and responsible professional men, who are willing to vouch for it. Our readers need have no hesitation in ordering from our advertisers, for the standing of every one has been thoroughly investigated. Offered advertisements are sometimes declined because we are not satisfied of the responsibility of the parties, or know nothing of the article offered, and sometimes they are held over a month until due enquiries and examinations can be made. We fully realize that we owe a duty first of all to our subscribers, and no one, with our knowledge and permission, shall be permitted to offer them a doubtful article.

